# Inferring intelligence of ancient people based on modern genomic studies

**DOI:** 10.1038/s10038-022-01039-8

**Published:** 2022-05-09

**Authors:** Kaisar Dauyey, Naruya Saitou

**Affiliations:** 1 Department of Genetics, School of Life Science, Graduate University for Advanced Studies (SOKENDAI), Mishima, Shizuoka 411-8540 Japan; 2 SAITOU Laboratory, National Institute of Genetics, Mishima, Shizuoka 411-8540 Japan

**Keywords:** Sequence annotation, Evolutionary biology

## Abstract

Quantification of ancient human intelligence has become possible with recent advances in polygenic prediction. Intelligence is a complex trait that has both environmental and genetic components and high heritability. Large-scale genome-wide association studies based on ~270,000 individuals have demonstrated highly significant single-nucleotide polymorphisms (SNPs) associated with intelligence in present-day humans. We utilized those previously reported 12,037 SNPs to estimate a genetic component of intelligence in ancient Funadomari Jomon individual from 3700 years BP as well as four individuals of Afanasievo nuclear family from about 4100 years BP and who are considered anatomically modern humans. We have demonstrated that ancient individuals could have been not inferior in intelligence compared to present-day humans through assessment of the genetic component of intelligence. We have also confirmed that alleles associated with intelligence tend to spread equally between ancestral and derived origin suggesting that intelligence may be a neutral trait in human evolution.

## Introduction

Intelligence is a complex phenotypic trait that has a large genetic component with high heritability [1]. Large-scale genome-wide association studies (GWAS) indicate that prediction of intelligence may be possible using genetic data [2]. The most powerful meta-analysis of GWAS based on 269,867 individuals identified several hundred genetic markers explaining up to 5.2% of variance in intelligence [3]. The assessment of cognition in that study was UK biobank fluid intelligence score derived from 13 questions focusing on memory, logic, verbal and numeric reasoning [4]. The genetic markers have been reported in the form of single-nucleotide polymorphisms (SNPs) spread across the whole human genome in high association with bidirectional effect in form of *Z*-score obtained through METAL software [5]. We have used those SNPs in our analysis based on classical assumption of single-nucleotide polymorphisms being the most common type of genetic variation in human genome and present in at least 1% of the global population [6].

Assessment of human intelligence and cognitive abilities is a challenging task. One of the challenges is lack of clear-cut definition of intelligence. Intelligence includes genetic and environmental components providing humans with multiple capacities of processing new information, linguistic as well as mathematical abilities, creativity that has been defined by more than two dozen definitions by American Psychological Association [7].

Quantification of cognitive abilities is a historically controversial issue. Intelligence quotient (IQ) tends to be a universal metric of neuropsychological abilities like mental speed, decision making, and problem-solving required for educational planning [8]. A large cohort of other tests exist like Peabody Picture Vocabulary test (a measure of children’s verbal intelligence) as well as scholarly aptitude tests like Scholastic Aptitude Test for colleges or Graduate Record Examination utilized by graduate schools in the United States. Previous research has demonstrated that performance on a subset of a particular test correlated with results on other parts of the test [9].

Intelligence assessment in ancient people like *Neanderthal* and *Australopithecus* has been attempted via medical imaging of fossilized skulls. Medical imaging used to reconstruct brain volumes of Australopithecus individuals living 3–4 million years (Myr) ago confirmed that anatomically modern humans (AMHs) have on average three times larger brain size [10]. Comparison of brain volumes in early and late AMHs (living 300,000–10,000 years ago) did not demonstrate significant difference in terms of total volume, but rather confirmed more complex structure in cortical areas with larger parietal lobe and cerebellum in more recent specimens [11]. The cortical areas more prominent in late AMH fossils compared to early samples are largely responsible for development of social tasks likely contributing to population growth and development of language as early as 50,000 years ago [12].

An alternative approach to fossil analysis termed “neuroarcheology” has utilized brain imaging of modern humans engaged in process of creating stone age tools in attempt to mimic ancient human brain activity [13]. Brain activity recorded during knapping of Acheulian handaxes dated ~1.75 Myr ago was significantly different from brain activity involved in making Oldowan tools dated about 2.6 Myr. Making more skillfully shaped Acheulian tools required activation of complex neural circuitry similar to brain activity observed during playing piano [14].

Anatomical measurements of brain structures, modeling cerebellar blood flow, mathematical modeling of brain size—body physiological parameters in ancient human species overall have demonstrated a trend of increasing brain sizes as well as higher metabolic neuronal activity suggesting an upward rise of human cognitive abilities in AMHs compared to *Neanderthal* and *Australopithecus* [15, 16]

The aim of this study was to assess a genetic component of intelligence in ancient individuals who are considered AMHs through genome analysis. Intelligence as a complex trait has likely been shaped by genetic variation in the course of evolution. We utilized 12,037 SNPs distributed across entire genome. Sex chromosomes weren’t included in our analysis since none of the X-chromosome and Y-chromosome SNPs have not reached the *p* value <5 × 10^−8^ threshold in the GWAS discovery phase [3]. We applied the derived polygenic scores (PGS) to 5 ancient genomes from ancient individuals (Table 1) including Funadomari Jomon individual discovered in Hokkaido, Japan with high sequencing coverage and peak depth of 48x (estimated age about 3700 years BP) [17]. Four other ancient genomes data originated from a nuclear family of four—a mother, a father, and their two sons from Afanasievo Culture discovered in modern Russia, who lived about 4100 thousand years BP (https://reich.hms.harvard.edu/datasets) [18].

**Table 1:** Ancient genomes used for polygenic estimation of intelligence

Sample name	Average coverage	Reference
Funadomari Jomon	48x	Kanzawa-Kiriyama et al. [16]
Afanasievo mother	21.2x	Wohns et al. [17]
Afanasievo father	25.3x	Wohns et al. [17]
Afanasievo son1	10.8x	Wohns et al. [17]
Afanasievo son2	25.8x	Wohns et al. [17]

Our analysis was aimed at elucidating a genetic component of intelligence in late AMHs (largely originating within 10,000 years ago) such as individuals from Jomon and Afanasievo cultures. The reason for that is the GWAS summary statistics obtained from present-day humans around the globe aligned to more archaic genomes like Denisovans and Neanderthal has a high likelihood of non-interpretable results due to considerable population divergence time (up to 170,000–700,000 years between Denisovans and present-day humans) [19]. We compared intelligence PGS derived from genomic data of ancient individuals (considered as AMHs) to 2504 present-day humans from the 1000 Genome Project Phase 3 [20]. We also inferred absolute IQ scores for ancient individuals compared to general population based on a genetic component of intelligence.

## Methods

### Selection of SNPs for polygenic scoring

Genetic markers of intelligence were obtained from a large-scale meta-analysis of GWAS on cognitive abilities with 269,867 participants from 14 European epidemiological cohorts [3]. Genome-wide significance (*p* < 5 × 10^−8^) in association with intelligence was confirmed for total number of 12,110 SNPs. Polygenic risk score prediction demonstrated that around 5.2% variation in intelligence can be explained by those SNPs. We estimated ancestral state of the majority of SNPs associated with intelligence by multiple alignment of reference genome of modern human (GRCH37) to bonobo, chimpanzee, gorilla, orangutan, gibbon, and macaque using “Ortheus” method implemented in ENSEMBLE database [21]. Fisher’s exact test of independence was used to assess any nonrandom association between ancestral state and effect on intelligence (Table 2).

**Table 2:** Ancestral state analysis of SNPs associated with intelligence

	Ancestral state		Total
	Ancestral	Derived	
Regression direction			
Positive	﻿2542	﻿3333	﻿ 5875
Negative	﻿2490	﻿3262	﻿ 5752
Total	5032	﻿6595	﻿11,627

### Calculating intelligence polygenic scores

We built PGS using 12,037 SNPs that reached genome-wide significance (*p* < 5 × 10^−8^) in the GWAS summary statistics. We utilized publicly available datasets from 1000 genome project phase 3 data (2504 individuals across global 26 populations) to construct PGS for each individual. The Funadomari Jomon genome sequence was selected for analysis due to high sequencing coverage (peak depth of 48x) considered as “the reference Jomon genome”. Four ancient European genomes with high-quality sequence belonging to Afanasievo culture were used for comparison as well (Table 1). Although the intelligence-associated SNPs have been identified in European populations we proceeded with evaluation of genetic component of intelligence in above mentioned ancient high-quality genomes in spite of estimated age of 3900–4100 BP.

We used PLINK version 1.9 [22] and R version 4.0.2 [23] to compute intelligence PGS. Data visualization was done through ggplot2 implemented in R [24]. Genetic intelligence scores were obtained by summing up the GWAS meta-analysis output beta regression coefficients identified for effective alleles in independent UK Biobank data subset for educational attainment replication. Each subject score was calculated as a sum of SNP effects considering number of effect allele presence (0, 1, or 2) multiplied by reported beta regression coefficients using polygenic risk score calculation [25].

We calculated genetic component of intelligence by constructing PGS through a linear model for each individual of the study cohort. Intelligence PGS PGS for each individual was defined in the form of:

${{{{{{{\mathrm{PGS}}}}}}}} = \beta _1x_1 + \beta _2x_2 + \ldots + \beta _kx_k + \beta _nx_n$, where $\beta _k$ represents per-allele beta coefficient of logistic regression for intelligence at SNP *k*, and $x_k$ based on allele dosage of 0, 1, or 2 for SNP *k* with total *n* number of SNPs included in PGS.

We used two subsets of SNPs from total 12,037 SNPs for PGS derivation: one set comprised of 9128 SNPs (*p* value threshold *p* < 5 × 10^−8^) as well as a smaller set of 1402 SNPs (*p* value threshold *p* < 4 × 10^−11^) replicated in an independent UK Biobank cohort and having top association in the original GWAS study. We decided to build two PGS based on different threshold of *p* values for SNPs reported in the original GWAS study according to generally accepted guidelines on performing PGS analysis through comparison of PGS and absolute IQ scores for ancient genomes to present-day humans [26]. Functions for calculating PGS and data visualization are available as R scripts on GitHub (https://github.com/Kays3/Ancient_intelligence.git).

### Statistical inference

The overall PGS of the intelligence data was tested for normality (Shapiro–Wilks’s test) and plotted assuming normal distribution. PLINK version 1.9 was used to extract the genotype data calculating eigen-values for principal component analysis (PCA), and building matrixes for computing genetic intelligence scores for each subject. Population structure demonstrated by PCA was built based on subset of 9128 SNPs and 1402 SNPs shared by modern and ancient human genomes. We also inferred absolute values of IQ for ancient individuals based on PGS results and compared them to a general human population mean of 100 and standard deviation (SD) of 15 [27] using open-access software designed for translating PGS into relevant absolute values of phenotypical traits [28].

## Results

We have built genotypes based on 12,037 SNPs highly associated with intelligence from genome sequences of 2504 individuals from 1000 Genome Project Phase 3. We also constructed two sets of genotypes comprised of 9148 SNPs and 1402 SNPs for genomes of Funadomari Jomon individual, Afanasievo family of four individuals as well as 1000 Genome Project subjects. Intelligence PGS constructed for total 2509 individuals were tested for normality using Shapiro–Wilk test. Intelligence PGS were indeed normally distributed (*W* = 0.99964, *p* value = 1.22 × 10^−5^).

**Fig. 1:**
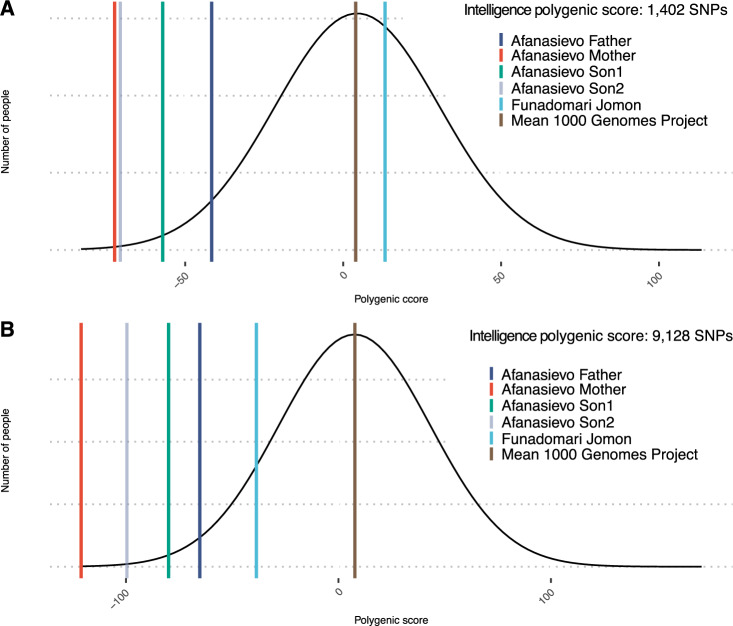
Intelligence polygenic scores in ancient individuals compared to modern humans. **A** PGS based on top significant 1402 SNPs out of 12,110 total SNPs (*p* value threshold *p* < 4 × 10^−11^) Afanasievo Mother *z* = −2.94, Afanasievo Son1. *z* = −2.36, Afanasievo Son2 *z* = −2.88, Afanasievo Father *z* = −1.77. Intelligence polygenic scores in Afanasievo individuals observed within 3 standard deviations (SD) from the mean of 1000 Genome Populations while Funadomari Jomon individual is above the mean, *z* = 0.33. **B** Funadomari Jomon individual, Afanasievo individuals PGS based on 9148 out of 12,110 total SNPs (*p* value threshold *p* < 5 × 10^−8^) extracted in common with 1000 Genome Populations. Afanasievo Mother *z* = −3.59, Afanasievo Son1 *z* = −2.45, Afanasievo Son2 *z* = −2.99, Afanasievo Father *z* = −2.04. Intelligence polygenic scores in Afanasievo individuals fell within 3 SD from the mean of 1000 Genome Populations while Funadomari Jomon individual is within 2 SD below the mean, *z* = −1.29

The PGS of intelligence based on highly significant 1402 SNPs (*p* value threshold *p* < 4 × 10^−11^) demonstrated Funadomari Jomon individual’s value within 1 SD above population mean of 1000 Genome Project (*z* = 0.34), while Afanasievo mother had a score lower than 2 SD of the mean (*z* = −2.94), Afanasievo father score was also below 2 SD from the mean (*z* = −1.77). Afanasievo sons scores were located between the maternal and paternal values where Son1 had a score of *z* = −2.36 and Son2 *z* = −2.88 (Fig. 1A).

The PGS of intelligence based on 9128 SNPs (*p* value threshold *p* < 5 × 10^−8^) placed Funadomari Jomon individual within 1 SD below population mean of 1000 Genome Project (*z* = −1.29), while Afanasievo mother had score lower than 2 SD of the mean (*z* = −3.59), Afanasievo father score was below 1 SD from the mean (*z* = −2.03). Afanasievo sons scores were located between the maternal and paternal scores where Son1 score *z* = −2.44 and Son2 with *z* = −2.99 (Fig. 1B).

**Fig. 2:**
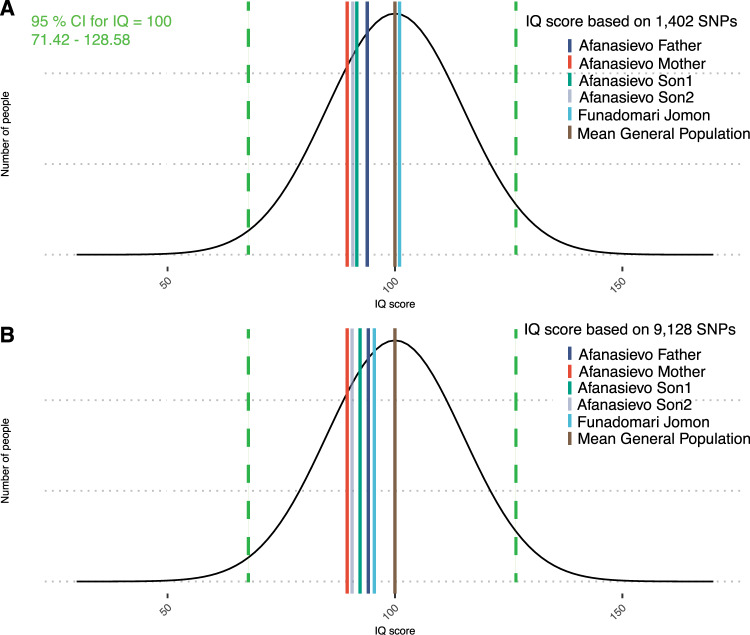
Absolute IQ values inferred from intelligence polygenic scores in ancient individuals compared to modern humans. **A** Absolute IQ values inference based on variance explained by intelligence polygenic score build from 1402 SNPs (*p* value threshold *p* < 4 × 10^−11^) demonstrated Funadomari Jomon individual’s IQ = 101 (95% CI = 72.58–129.74), while Afanasievo mother’s IQ = 89 (95% CI = 60.96–118.12), Afanasievo father IQ = 94 (95% CI = 65.2–122.36). Afanasievo sons scores were located between the maternal and paternal scores where Son1 score had IQ = 92 (95% CI = 63.17–120.3) and Son2 IQ = 90 (95% CI = 61.54–118.7). Variance explained by intelligence polygenic score (*R*^2^ = 5.2%), mean IQ = 100 with SD = 15 (95 % CI = 71.42–128.58) in modern human general population. **B** Absolute IQ values inference based on variance explained by intelligence polygenic score build from 9128 SNPs (*p* value threshold *p* < 5 × 10^−8^) demonstrated Funadomari Jomon individual’s IQ = 95 (95% CI = 66.88–124.04), while Afanasievo mother’s IQ = 87 (95% CI = 57.96–115.12), Afanasievo father IQ = 93 (95% CI = 64.3–121.46). Afanasievo sons scores were similarly located between the maternal and paternal scores where Son1 had IQ = 91 (95% CI = 62.87–120.02) and for Son2: IQ = 89 (95% CI = 60.96–118.12)

Absolute IQ score inference based on variance explained by intelligence PGS (5.2%) with mean of the trait of 100 and SD of 15 demonstrated following scores for ancient individuals: for the 1402 SNPs PGS Funadomari Jomon individual IQ = 101 (95% CI = 72.58–129.74), while Afanasievo mother’s IQ = 89 (95% CI = 60.96–118.12), Afanasievo father IQ = 94 (95% CI = 65.2–122.36). Afanasievo sons scores were located between the maternal and paternal scores where Son1’s IQ = 92 (95% CI = 63.17–120.3) and Son2’s IQ = 90 (95% CI = 61.54–118.7) (Fig. 2A).

Absolute IQ score estimates for PGS based on 9128 SNPs demonstrated Funadomari Jomon individual’s IQ = 95 (95% CI = 66.88–124.04), while Afanasievo mother’s IQ = 87 (95% CI = 57.96–115.12), Afanasievo father IQ = 93 (95% CI = 64.3–121.46). Afanasievo sons scores were similarly located between the maternal and paternal scores where Son1 had IQ = 91 (95% CI = 62.87–120.02) and for Son2: IQ = 89 (95% CI = 60.96–118.12) (Fig. 2B).

**Fig. 3:**
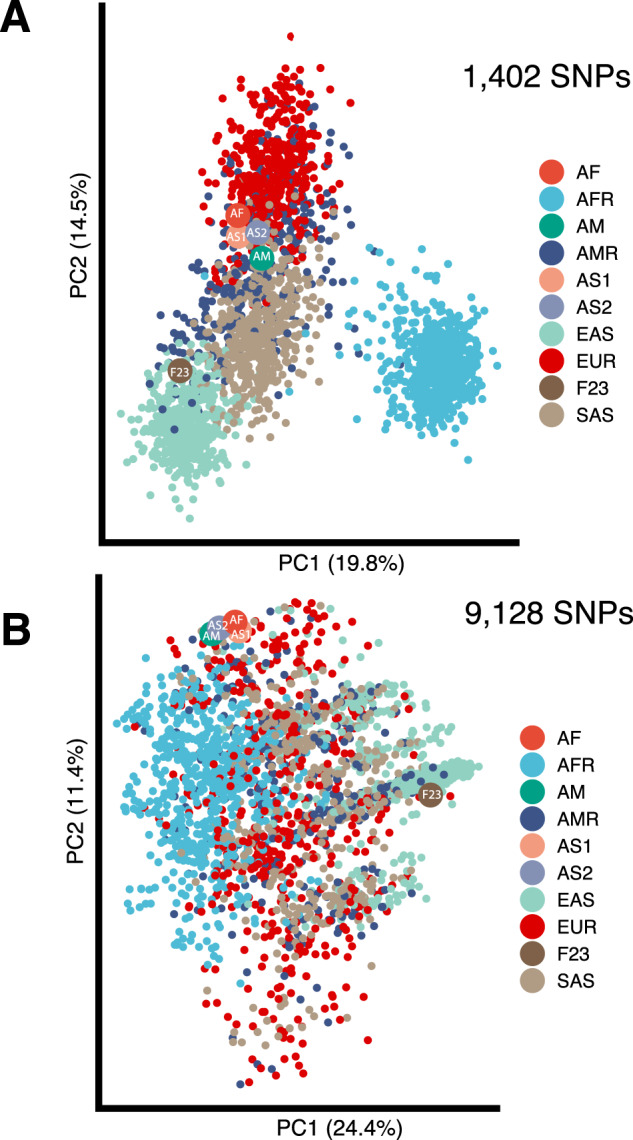
Intelligence-associated genetic relationship based on principal component analysis between ancient and modern humans. **A** Principal component analysis based on 1402 top significant SNPs out of 12,110 SNPs (*p* value threshold *p* < 4 × 10^−11^) shared by ancient individuals and subjects from 1000 Genomes Project. Ancient individuals including Funadomari Jomon (F23), Afanasievo family—Mother (AM), Father (AF), Son1 (AS1), Son2 (AS2) and modern humans defined in accordance with the groupings in the 1000 Genomes Project: European (EUR), admixed American (AMR), East Asian (EAS), African (AFR) and South Asian (SAS); **B** principal component analysis based on 9128 SNPs out of 12,110 SNPs (*p* value threshold *p* < 5 × 10^−8^) in high association with intelligence

PCA based on 1402 SNPs demonstrated close genetic relationships between ancient Funadomari Jomon individual to modern East Asian populations while Afanasievo family individuals clustered with modern European populations from the 1000 Genome Project (Fig. 3A). PCA based on 9128 SNPs did not reveal any clear population structure with Funadomori Jomon individual clustered in proximity to East Asian populations compared to Afanasievo family (Fig. 3B).

We assessed ancestral state of 12,037 SNPs used for PGS construction in 1000 Genome Populations through alignment with six primate genomes including bonobo, chimpanzee, gorilla, orangutan, gibbon, and macaque using “Ortheus” method implemented in ENSEMBLE database [21]. The alignment results between ancestral species and GWAS summary statistics used for polygenic scoring was available only for 11,627 SNPs (96.6%) accessible from ENSEMBLE database. SNPs contributing to positive effect and negative effect on intelligence demonstrated no association with derived state (*p* value = 0.985, OR with 95% CI = 1.00 [0.93–1.10]) based on Fisher exact test (Table 2). Ancestral state analysis of SNPs highly associated with intelligence did not demonstrate any significant differences suggesting that genomic regions contributing to intelligence PGS are present not only in modern humans, but in primates as well.

## Discussion

We have demonstrated first ever insight into genetic component of intelligence through PGS in ancient individuals from around 3700–4100 BP. The performed calculations indicate a possibility that people living on the territory of modern Hokkaido and Russia in that period being not less intelligent than modern humans. Absolute IQ values inferred from PGS in Afanasievo individuals and Funadomari Jomon individual tends to be within the 95% range of mean general human population suggesting similarity of intelligence of humans living 3700 BP and modern humans. Although intelligence PGS of Afanasievo family tend to fluctuate on the lower tail of normal distribution of the scores of 1000 Genome project these scores translate to absolute IQ values within mean of general population given the low variance explained by intelligence PGS (*R*^2^ = 5.2%).

We used two different *p* value thresholds for constructing PGS of intelligence, since there is no clear consensus on how selection of SNPs may affect the predictive power of the analysis. Previous work on PGS of intelligence demonstrated that different thresholds may actually have association with particular aspects of cognition like memory or verbal intelligence [29].

Previous studies on PGS prediction confirmed lower applicability and reproducibility of the majority of GWAS reported in global populations due to the fact that most data used in the discovery phase came from people of European descent [30, 31]. However, recent development in derivation of absolute trait values from PGS confirmed potential clinical utility and rationale of polygenic prediction in context of complex traits and clinical decision making [32, 33].

Even though there is a possibility that SNPs associated with cognition may have lower predictive abilities when applied to non-Europeans, there have not been any other studies reporting intelligence prediction of ancient individuals through genetic data to our knowledge. This analysis is an example of application of GWAS findings toward assessment of cognitive abilities in individuals living around 4000 years ago. Previous studies on polygenic prediction of height as well disease risk in ancient DNA confirmed similar predictive power in ancient humans to modern individuals [34, 35].

Modern concept on intelligence measured by IQ holds on principle of dual contribution of genetic and environmental components (socioeconomic aspects, medical care) forming essential cognitive functions. IQ measures have been implicated with survival, adaptation to environment, and mental functioning [8]. Digital genomic biobank DNA.Land as well as various genetic applications like GenePlaza, 23andMe previously reported polygenic prediction of a number of complex traits including intelligence based on GWAS findings [36, 37]. Although the predictions have the potential to elucidate individual traits in comparison to massive digital databanks, a small percent of genetic contribution to the traits is still the most important limiting factor in wider applicability of any predictions [38].

DNA.Land platform has previously demonstrated evaluation of intelligence PGS using GWAS findings based on 72 SNPs [2]. The variance of intelligence explained by polygenic scoring in DNA.Land study was about 4.8%. Although 12,110 SNPs we used in our study only explain about 5.2% variance in intelligence through PGS, large number of those SNPs have been mapped to protein coding and non-coding DNA elements highly associated with cognitive functions and mental disorders [3]. Likely such a modest increase in predictive power of intelligence based solely on genetic factors suggests a need for alternative intelligence prediction tools incorporating environment and socioeconomic factors.

A common approach in studying quantitative traits like intelligence in humans has been based on monozygotic and dizygotic twins [39]. Previous studies on three-dimensional brain mapping in twins supported correlation between gray-matter volumes in genetically identical twins and high heritability for brain areas responsible for IQ, speech, and language [40, 41]. High heritability of intelligence has also been criticized due to overlap of cognitive ability measurements with various factors like presence of IQ statistics, socioeconomic influence, and other environmental influences [42].

Intelligence as a phenotypic trait with underlying effects of DNA polymorphism has been likely shaped by evolutionary processes. Majority of mutations in genes affecting underlying cognition used in this study tend to interact in extremely complex networks with higher activity in hippocampal as well as somatosensory neurons [3]. Since not only humans, but primates have active neurogenesis in those brain areas [43] we hypothesize that genetic contribution to intelligence through mutations are shared to some extent with human ancestral species. High abundance of shared SNPs related to intelligence in primates and humans observed in our study may suggest that most mutations in genomic regions associated with intelligence of ancient humans and their ancestors are in line with neutral theory of evolution [44]. We have demonstrated conserved state of half of causative SNPs in primates and humans (Table 2). Since the ancestral state inference was done in relation to primates, there is no clear boundary between alleles contributing to higher intelligence being more common in modern humans than in ancestral species.

We demonstrated that genomic data from ancient individuals can be used to evaluate a genetic component of intelligence. Funadomari Jomon as well as Afanasievo family individuals demonstrated intelligence PGS as well as IQ scores in line with modern humans. DNA evidence may indicate a possibility of intelligence being a neutral trait in human evolution suggesting that ancient individuals living 3700–4100 years BP could have been as intelligent as modern humans.
